# Proliferative reactive gliosis is compatible with glial metabolic support and neuronal function

**DOI:** 10.1186/1471-2202-12-98

**Published:** 2011-10-10

**Authors:** Félix R Vázquez-Chona, Alex Swan, W Drew Ferrell, Li Jiang, Wolfgang Baehr, Wei-Ming Chien, Matthew Fero, Robert E Marc, Edward M Levine

**Affiliations:** 1 Department of Ophthalmology and Visual Sciences, John A. Moran Eye Center, University of Utah, 65 Mario Capecchi Dr., Salt Lake City, UT 84132, USA; 2 Clinical Research Division, Fred Hutchinson Cancer Research Center, 1100 Fairview Avenue North, Seattle, WA 98109, USA

**Keywords:** reactive gliosis, retinal degeneration, neuronal degeneration, GFAP, Müller glia, p27Kip1, CDKN1B, intermediate filaments

## Abstract

**Background:**

The response of mammalian glial cells to chronic degeneration and trauma is hypothesized to be incompatible with support of neuronal function in the central nervous system (CNS) and retina. To test this hypothesis, we developed an inducible model of proliferative reactive gliosis in the absence of degenerative stimuli by genetically inactivating the cyclin-dependent kinase inhibitor *p27^Kip1 ^*(*p27 *or *Cdkn1b*) in the adult mouse and determined the outcome on retinal structure and function.

**Results:**

p27-deficient Müller glia reentered the cell cycle, underwent aberrant migration, and enhanced their expression of intermediate filament proteins, all of which are characteristics of Müller glia in a reactive state. Surprisingly, neuroglial interactions, retinal electrophysiology, and visual acuity were normal.

**Conclusion:**

The benign outcome of proliferative reactive Müller gliosis suggests that reactive glia display context-dependent, graded and dynamic phenotypes and that reactivity in itself is not necessarily detrimental to neuronal function.

## Background

In response to neural pathologies, glia display reactive properties associated with wound healing including cellular hypertrophy, proliferation, migration and cytokine release [1-4]. In mammalian CNS and retina, reactive glia contribute to neural tissue repair [5-10] but also to neural dysfunction, scar formation, abberant neural rewiring, and vascular remodeling [1-3,11,12], ultimately exacerbating neuronal degenerations [11,13]. Defining the components of reactive gliosis that are detrimental to neuronal survival and tissue integrity is an important goal but difficult to achieve. Animal models of reactive gliosis also induce neuronal cell death, microglial reactivity, inflammatory responses or tissue damage [1,3,4,14]. An alternate approach to explore glial reactivity and neuronal metabolism, physiology and function is to develop genetically inducible models of reactivity in the absence of gross degenerative cues.

Two hallmarks of reactive glia are proliferation and enhanced intermediate filament expression. Both are associated with opposing properties: neuroprotection and degeneration. Experimental models and gene inactivation studies implicate upregulation of intermediate filament expression in the formation of hypertrophic glial processes. Glial hypertrophy helps maintain the structural integrity of the CNS by filling the space where neurons die and by restoring damaged protective barriers [9,15,16]. However, intermediate filaments are abundant in glial scars which are known to impede axonal regeneration [17,18]. Chronic upregulation of intermediate filament expression is also correlated with glial metabolic dysfunction and altered neuronal electrophysiology [12,19-21]. The role of glial proliferation is similarly perplexing. Genetic ablation of proliferating glia worsens neurodegeneration [5,6] while pharmacological inhibition of glial proliferation enhances neuronal survival and function [14,22]. Given these complexities, more precise dissections of the links between glial reactivity and progressive neurodegeneration are needed.

The cyclin-dependent kinase inhibitor p27 is one such link. It is expressed in many adult glial populations including Schwann cells, cortical astrocytes, spinal cord astrocytes, oligodendrocytes, and retinal Müller glia [23-27]. In germline p27-deficient mice (*p27^-/-^*), adult glia can display hallmarks of reactive gliosis [24-26,28]. In the wild-type retina, quiescent Müller glia normally do not express the intermediate filament glial fibrillary acidic protein (GFAP), but Müller glia in *p27^-/- ^*mice express high levels of GFAP and in some instances migrate into the subretinal space [24,26]. This behavior is enhanced by the combinatorial inactivation of *p27 *and the cyclin-dependent kinase inhibitor *p19^Ink4d ^*[29]. Müller glial reactivity and abnormal retinal electrophysiology in *p27^-/- ^*mice may partly arise from developmental dysregulation as p27 is critical for neural development and glial differentiation [24,26,30-33]. Even so, CNS and retinal trauma models support a role for p27 in maintaining mature glial cells in a quiescent, supportive state. After acute trauma, cortical astrocytes, spinal cord astrocytes and retinal Müller glia downregulate p27, upregulate GFAP and re-enter the cell cycle [14,25,27,34]. Thus p27 appears to be a negative regulator of two classic indices of reactive glia: GFAP upregulation and proliferation. This implies that selective inactivation of *p27 *could trigger neural remodeling and reprogramming defects in an otherwise normal milieu.

To modulate discrete reactivity indices in the absence of other degenerative stimuli, we induced intermediate filament GFAP upregulation, migration, and proliferation in adult Müller glia by inactivating p27 using a tamoxifen-regulated, Cre-loxP system [35,36]. This approach bypassed the developmental requirement for p27 [24,26] as well as the complexities and broad effects of experimentally induced degeneration [1,3,4,14]. To address the significance of enhanced discrete reactivity on neuronal survival and function, we surveyed metabolism, retinal electrophysiology, and visual acuity. Contrary to our expectations, proliferative and GFAP-expressing Müller glia did not significantly impair retinal metabolism, electrophysiology, or visual function. Thus, our genetic model and the p27 pathway offer a new platform to explore how environmental factors involved in neuronal cell stress, microglial activation, inflammatory responses, or blood barrier damage contribute to the transition of resident glia from a supportive to detrimental state.

## Results

### Inducible model of p27 deficiency in adult mice

**Figure 1:**
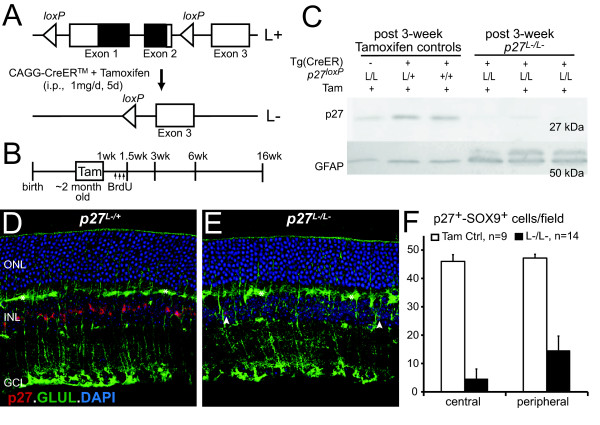
**Inducible model of *p27^Kip1 ^*inactivation in adult mice**. (A) The p27 coding region was conditionally targeted by generating mice harboring *LoxP *sites at the *p27 *locus (*p27^L+^*) and expressing a tamoxifen-regulated Cre recombinase under the control of the chimeric chicken beta-actin promoter, *CAGG*::CreER™. (B) Experimental design: Tamoxifen was administered by single daily intraperitoneal injections (i.p.) for 5 consecutive days in 2 month-old mice. For proliferation analysis, BrdU was injected once daily for three consecutive days starting 8 days after the first tamoxifen injection. All timepoints were calibrated to the start of tamoxifen exposure. (C) Western blots for p27 and the marker of glial reactivity GFAP. Each lane contains 15 μg of protein from individual retinas. The blot was cut at ~35 kDa: portions with the lower and higher molecular weight bands were stained for p27 and GFAP, respectively. (D and E) Immunostaining against p27 and the Müller glial marker Glutamine Synthetase (GLUL). Micrographs of control (*p27^L-/L+^*) and experimental animals (*p27^L-/L-^*) six weeks after the start of tamoxifen injections. Asterisks indicate blood vessels. Arrowheads denote Müller glia that failed to undergo p27 inactivation. (F) Inactivation efficiency was measured by comparing the number of p27^+ ^cells colocalizing with the glial nuclear marker SOX9. Data are expressed as the mean ± SD. Abbreviations: ONL, outer nuclear layer; INL, inner nuclear layer; RGC, retinal ganglion cell layer; p27, cyclin-dependent kinase inhibitor CDKN1B; GFAP, glial fibrillary acidic protein; GLUL, glutamine synthetase; SOX9, SRY-box containing gene 9; and DAPI, 4', 6-diamidino-2-phenylindole.

We conditionally targeted the p27 coding region in adult mice harboring *LoxP *sites at the *p27 *locus (*p27^L+^*), and expressing a tamoxifen-regulated Cre recombinase under the control of the chimeric chicken beta-actin promoter, *CAGG*::CreER™ (Figure 1A and 1B) [35,36]. Since high levels of tamoxifen can be toxic to retinal cells [37], expressing CreER™ with a strong promoter facilitated a tamoxifen dose that did not cause neuronal cell death or reactive gliosis (50 μg tamoxifen/gbw, see methods; Figure 1C and analyses below). Importantly, this dose greatly reduced p27 expression in targeted mice (*p27^L-^*; Figure 1C-F). Inactivation efficiency was higher in the central retina than in the peripheral retina (90.0 ± 7.3 and 68.5 ± 10.7%; Figure 1F and Additional file 1). These results are consistent with previous recombination studies in adult retina and might reflect variability in CreER™ expression or tamoxifen accesibility [36,38]. Müller glia failing to undergo *p27 *inactivation allowed us to discriminate between cell autonomous and non-cell autonomous effects of *p27 *inactivation. While genetic deletion of *p27 *can also occur in neurons expressing CreER™, this was likely inconsequential as mature retinal neurons do not express p27 [24,26]. Thus, our model of p27 inactivation displayed optimal experimental conditions to define the role of p27 in Müller glial reactivity.

### p27-deficient Müller glia upregulate intermediate filaments

**Figure 2:**
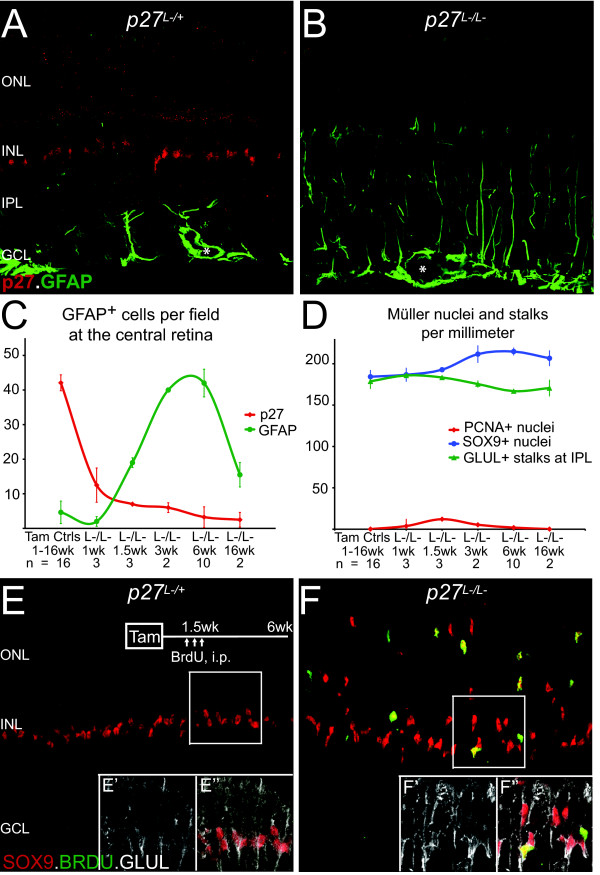
**Inducible *p27 *inactivation results in enhanced intermediate filament GFAP expression, glial cell-cycle entry, and glial nuclear migration**. (A and B) Immunostaining for p27 and the marker of glial reactivity GFAP. Micrographs of tamoxifen control (*p27^L-/L+^*) and experimental (*p27^L-/L-^*) retinas six weeks after the start of tamoxifen injections. Asterisks indicate blood vessels. (C and D) Quantitation of Müller glia becoming reactive (GFAP^+^) and entering cell cycle (PCNA^+^) as well as the total number of Müller glial nuclei (SOX9^+^) and stalks (GLUL^+^). Curves were generated using the Smooth marked scatter option in Excel™. (E-F) Bromodeoxyuridine (BrdU) injections followed by a six week chase to reveal the fate of dividing cells. (E-F insets) Higher magnification showing colocalization with glial marker GLUL. Data are expressed as the mean ± SD. Abbreviations: PCNA, Proliferating cell nuclear antigen.

Since upregulation of the intermediate filament GFAP is a hallmark of glial reactivity, we examined GFAP immunoreactivity in retinas from *p27^L-/L- ^*mice and tamoxifen control mice (Figure 1C). Tamoxifen control retinas displayed two distinct patterns of GFAP expression. In the central retina, astrocytes located at the ganglion cell layer expressed GFAP (Figure 2A). At the far periphery, radial processes expressed GFAP that colocalized with Müller glial markers (Additional file 1). The patterns of GFAP expression in tamoxifen control retinas were consistent with those previously observed in wild-type retinas [39,40]. After *p27 *inactivation, western blots revealed enhanced expression of a 50-kDa GFAP band and of a lower molecular weight band (Figure 1C) which correlated with increased GFAP immunoreactivity in Müller glia both in the central and peripheral retina (Figure 2A and 2B; Additional file 1). Temporal and spatial analyses of GFAP-immunoreactivity revealed three distinct patterns of expression (Figure 2C). Seven days after the start of the *p27 *inactivation protocol, only a small percentage of Müller glia (5.5 ± 1.5%) displayed GFAP immunoreactivity restricted primarily to the end feet at the ganglion cell layer. Three to six weeks after inactivation, almost all Müller glia (91.9 ± 7.3%) displayed GFAP immunoreactivity throughout their cell bodies. Nearly four months after inactivation, the number of GFAP^+ ^Müller glia (34.4 ± 7.9%) and the extent of GFAP immunoreactivity throughout the cell body decreased. We also observed that a small percentage of p27^+ ^Müller glia expressed GFAP (Additional file 1, arrows), which suggests that *p27 *inactivation might influence cells through a non-autonomous mechanism. However, the vast majority of GFAP expression was observed in p27-deficient Müller glia (Figure 2B). We also found upregulation of the intermediate filaments vimentin and nestin in p27-deficient Müller glia (Additional file 1).

### p27-deficient Müller glia reenter the cell cycle

Müller glia do not proliferate under normal conditions in part due to the presence of cell cycle inhibitors such as p27 [24,25]. After conditional *p27 *inactivation, however, cells reentered the cell cycle as indicated by the upregulation of proliferative markers PCNA, pHH3, and MCM6 (Figure 2D and Additional file 2) as well as bromodeoxyuridine (BrdU) labeling (Figure 2E and 2F). BrdU injections followed by a six week chase revealed that BrdU colocalized with Müller glial markers SOX9 and glutamine synthetase (GLUL, Figure 2F) and not with photoreceptor or bipolar markers (Recoverin, OTX2, PKC-alpha). While the number of SOX9^+^-GLUL^+ ^nuclei increased by 15.8% (p <0.00003) the number of GLUL^+ ^stalks at the inner plexus layer decreased by 6.1% (p < 0.006) (Figure 2D). Increased GLUL^+ ^soma density with decreased stalk density was confirmed with ultrathin radial and oblique sections suggesting that proliferative Müller glia can retract their stalks before dividing and neither they or their daughters regrow stalks after dividing (Additional file 3). These data indicate that *p27 *inactivation in Müller glia was sufficient to induce cell cycle entry with a low level of proliferation.

SOX9 and GLUL immunoreactivity also revealed that p27-deficient Müller glia displaced their nuclei toward the photoreceptor layer (Figure 2F and Additional file 3). The displacement of Müller glial nuclei occured one week after the start of *p27 *inactivation and the ectopic location was irreversible. It is unclear whether the nuclear displacement reflects migration of Müller glia or simply interkinetic nuclear migration. Evidence supporting migratory-like behavior is the presence of focal and limited extension of GFAP^+ ^Müller glial endfeet into the photoreceptor segments (Additional file 4, arrows); but unlike *p27^-/- ^*retina [24,26], *p27^L-/L- ^*retina displayed continuous outer limiting membrane as seen by the Müller glia microvilli marker CD44 (Additional file 4). In sum, induced p27 deficiency results in adult Müller glia adopting classic indices of proliferative reactive gliosis: intermediate filament upregulation, cell cycle entry, and migratory-like behavior.

### Reactive p27^L-/L- ^Müller glia provide homeostatic metabolic support

**Figure 3:**
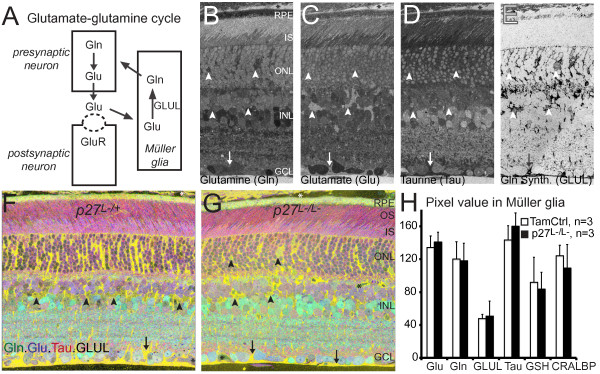
**Reactive *p27^L-/L- ^*Müller glia maintain neuronal metabolic support**. (A) Schematic of neuron-glia metabolic interaction via the glutamate-glutamine cycle. (B-E) Differential metabolite content of cells in *p27^L-/L- ^*retina visualized and quantified using computational molecular phenotyping (CMP). Plasticized tissue was serially sectioned at 200 nm, probed with specific anti-hapten IgGs, and visualized with silver-intensification of 1.4 nm gold granules coupled to species-specific secondary IgGs. Each metabolic map was captured as a high-resolution, monochrome image. (F-G) Metabolic mapping of tamoxifen control and experimental retina at the peak of GFAP expression (6 weeks after tamoxifen injections). Red-green-blue channels represents taurine-glutamate-glutamine mapping highlighted with GLUL as an alpha-channel. RGB images with alpha channels were prepared with Adobe Photoshop CS3. The yellow background is the distinctive taurine-glutamine signature of Müller glia. The pink and red compartments in the photoreceptor outer segments (OS) and inner segments (IS) contain distinct taurine-glutamate-glutamine mixtures. While various blue-to-azure cells in the interneuron layer (INL) and ganglion cell layer (GCL) are neurons with distinctive glutamate-glutamine mixtures. (H) Metabolite pixel value in Müller glia was extracted using the GLUL signal. Data are expressed as the mean ± SD. There was no statistically significant difference between tamoxifen control animals (n = 3) and experimental *p27^L-/L- ^*animals (n = 3). Asterisks, arrows and arrowheads indicate blood vessels, endfeet and somas, respectively. Additional abbreviations: CRALBP, cellular retinaldehyde-binding protein; GSH, glutathione.

The ability of reactive glia to maintain metabolic support and neuronal function is unclear as reactive glia often downregulate metabolic enzymes during neuronal degenerations [4,11,21,41]. We visualized and quantified the metabolic support that glia offer both excitatory and inhibitory neurons--glutamate and γ-Aminobutyric acid (GABA) uptake and their ultimate recycling into glutamine--using computational molecular phenotyping (CMP, Figure 3A). CMP measures metabolite concentration in N-dimensional space at the single cell level [12,21,42]. Molecular mapping on 200 nm sections with silver intensification revealed the spatial distributions of glutamate, glutamine, and glutamine synthetase during the peak of GFAP upregulation (6 weeks post-inactivation, Figure 3B-E). The ability of p27-deficient Müller glia to metabolize glutamate is so efficient that their somas and their endfeet displayed low levels of glutamate (Figure 3C, arrow heads and arrows). Metabolic maps representing taurine, glutamate and glutamine signals into red-green-blue channels revealed the compartmentalization of glutamate in excitatory neurons such as photoreceptors, bipolar cells and ganglion cells (Figure 3F and 3G, blue to purple hues). In these metabolic maps, the highly conserved taurine-glutamine signature of Müller glia (the yellow background) [12,21,42] is maintained in *p27^L-/L- ^*retina. Metabolite pixel values extracted using either the glutamine synthetase signal as a mask or formal multi-channel clustering [42] revealed that p27-deficient Müller glia maintained statistically inseparable levels of glutamate and glutamine compared to tamoxifen control retinas (Figure 3H). Additional markers of glial function including osmoregulation (taurine), redox function (glutathione), retinoid binding (cellular retinaldehyde-binding protein, CRALBP), and GABA recyling (GABA) were also unchanged (Figure 3H and Additional file 3E). Metabolite concentrations and distributions for photoreceptors and retinal pigmented epithelium were comparable in tamoxifen control and *p27^L-/L- ^*retinas. The p27-deficiency did not alter the levels of metabolites and enzymes important for retinal homeostasis and neuronal function.

### Normal electrophysiology in retinas with reactive p27^L-/L- ^Müller glia

**Figure 4:**
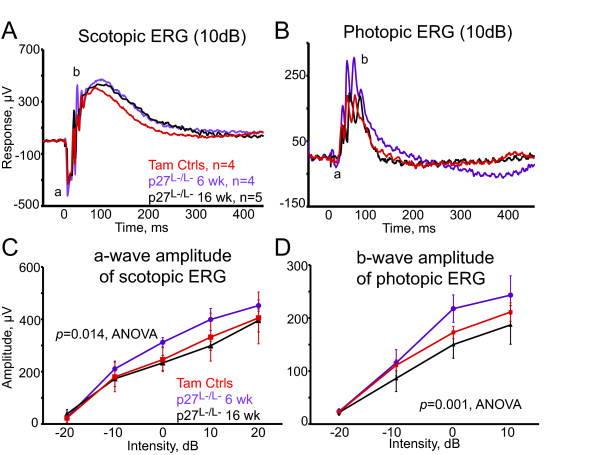
**Normal electrophysiology in retinas with reactive *p27^L-/L- ^*Müller glia**. Electroretinographic analysis was performed in experimental *p27^L-/L- ^*and control mice during the peak of GFAP upregulation and during chronic GFAP expression (6 and 16 weeks post-inactivation). During these time periods the Müller glia nuclei migrated from their normal position (mid-inner retinal layers) toward the photoreceptor layer. Four to five mice were analyzed for each condition. (A and B) Representative scotopic and photopic electroretinogram (ERG) responses evoked by 10 decibel (dB) light flashes were recorded after dark adaptation and light adaptation, respectively. The fast components of the a- and b-waves reflect the functional integrity of photoreceptor and bipolar cell signaling, respectively. The slow components of the b-wave reflect Müller glial conductance. (C and D) Wave amplitudes were averaged from 4-5 mice per condition and shown as function of stimulating light intensity. Data are expressed as the mean ± SD.

Several studies suggested that reactive gliosis alters neuronal responsivity well before any indication of neuronal cell death [12,19-21]. We therefore examined retinal function by electroretinography (ERG) during the peak of GFAP upregulation and during chronic GFAP expression (6 and 16 weeks post-inactivation). During these time periods the Müller glia nuclei migrated from their normal position (mid-inner retinal layers) toward the photoreceptor layer (Figure 2F and 3G). ERGs of tamoxifen control mice were essentially identical to those of adult wild-type mice. The *p27^L-/L- ^*a- and b-waves, reflecting the functional integrity of photoreceptor and bipolar cell signaling [43], were also normal under scotopic (rod-system) and photopic (cone system) conditions (Figure 4A and 4B). The small increase in *p27^L-/L- ^*b-wave amplitude and time-to-peak (Figure 4C and 4D) might be caused by decreased net potassium channel conductance in Müller glia [44,45]. However, the distribution and immunoreactivity level of the glial potassium channel Kir4.1 appeared normal in the *p27^L-/L- ^*retina (Additional file 4C and 4D), suggesting the possibility of increased impedence in Müller glia. Unlike the *p27^-/- ^*retina [32,33], *p27^L-/L- ^*retina did not exhibit pathologies characteristic of retinal dysfunctions such as reduced, delayed, truncated, or inverted ERG signals [46].

### Normal visual acuity and function in mice with reactive p27^L-/L- ^Müller glia

**Figure 5:**
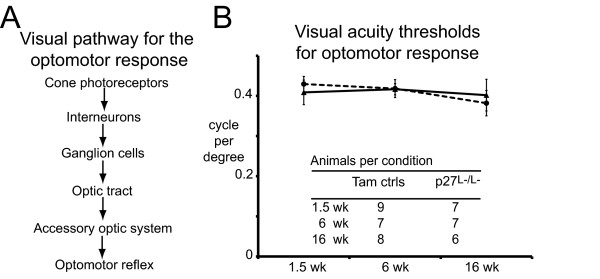
**Normal visual acuity in *p27^L-/L- ^*mice using the optomotor response**. (A) Known visual pathways interrogated by the optomotor response test. (B) Optomotor tracking response to moving gratings was measured using a virtual optomotor system. Spatial frequency thresholds that elicited an optomotor response between tamoxifen control and experimental animals during the peaks of Müller glial proliferation and GFAP upregulation, and during chronic GFAP expression (1.5, 6 and 16 weeks post-inactivation, respectively). A sine wave grating was drawn on a virtual cylinder projected on monitors and the cylinder was rotated to test each. The value for each animal represents the average of both eyes. Data are expressed as the mean ± SD.

We next tested photopic visual acuity using the optomotor response that evaluates the impact not only of Müller glial reactivity but also of reactive glia along the accessory optic system (Figure 5A), such as oligodendrocytes in the optic nerve, which normally express p27 (Additional file 4). Spatial frequency thresholds that elicited an optomotor response were not statistically different between tamoxifen control and experimental animals during the peaks of Müller glial proliferation and GFAP upregulation, or during chronic GFAP expression (1.5, 6 and 16 weeks post-inactivation respectively; Figure 5B). The spatial frequency threshold of the optomotor response in tamoxifen control and experimental animals (0.418 ± 0.027 cycles per degree) was comparable to previously published values in mice [47].

## Discussion

### p27 is a negative regulator of proliferative Müller glial reactivity

The hypothesis that p27 is a key modulator of glial plasticity is supported by our finding that inducing p27-deficiency is sufficient to promote proliferative Müller reactive gliosis in adult retina [24-27,34]. As a cell cycle inhibitor, p27 modulates glial proliferation and consequently p27 can modulate the potential of glial cells to regenerate neural tissue and to form scars [48]. In mammalian retinal degenerations, Müller glia may fail to re-enter the mitotic cycle because of persistent p27 expression [48]. In contrast, Müller glia and astrocytes adjacent to traumatic injuries downregulate p27 resulting in glial proliferation that contributes to scar formation [34]. The absence of neurogenesis and scar formation after our conditional *p27 *inactivation suggests that while decreased p27 activity modulates glial proliferation, transitioning to a neurogenic state or scar forming phenotype must be determined by additional signaling mechanisms. Indeed several groups found that addition of neurogenic factors are required to guide proliferating glia into a neurogenic state [24,25,48,49]. Our conditional *p27 *inactivation also yielded nonproliferative phenotypes including Müller glial nuclear migration, cytoplasmic process extension, and increased intermediate filament content. These phenotypes are consistent with data suggesting that the impact of p27 activity extends beyond cell cycle regulation, possibly by modulating transcription, cell fate, cell migration, or cytoskeletal dynamics [30,31]. The issue of whether the nonproliferative changes are the direct result of p27 deficiency or a secondary response to cell cycle reentry will be addressed in future studies; however, it is clear that p27 levels have broad effects on the outcome of glial reactivity even in the absence of degenerative cues. Consequently the p27 pathway represents a prime target to facilitate glial-based regeneration and to modulate glial scar formation.

### Reactive gliosis displays context-dependent, graded and dynamic phenotypes

**Figure 6:**
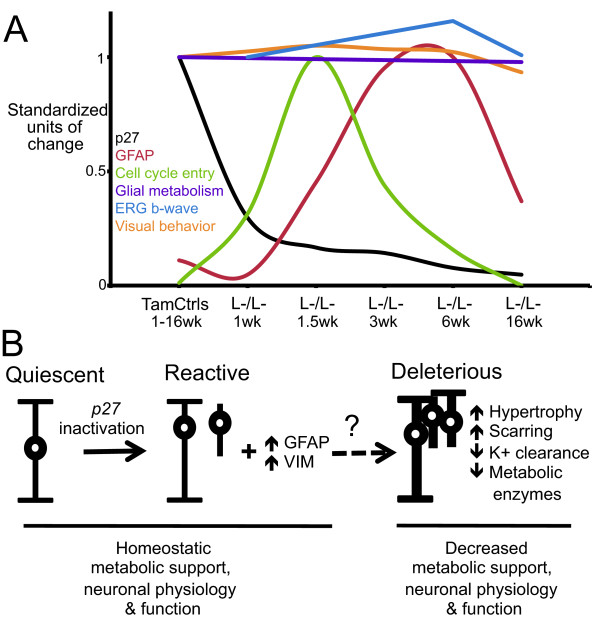
**Summary and model**. (A) Comparison of retinal glial and neuronal responses to *p27 *inactivation. Data values were standardized for cross comparison. (B) Model of *p27 *inactivation effect on glia and neuronal function. Conditional *p27 *deficiency results in cell cycle entry and soma displacement, as well as in enhanced intermediate filament expression (e.g., GFAP and VIM). Surprisingly, these reactive changes are compatible with homeostatic neuronal metabolism, electrophysiology and function. The benign impact of inducible proliferative reactive gliosis in *p27^L-/L- ^*mice raise the hypothesis that the transition from supportive to deleterious is determined by external factors signaled from either neuronal cell stress, microglial reactivity, inflammatory response, blood barrier damage, or neuronal cell death.

Our genetic model of proliferative reactive gliosis is one of several studies using gene targeting approaches to define the role of specific pathways in reactive gliosis (reviewed in [16,50,51]). The general theme from genetic dissection studies is that reactive gliosis is a multifaceted event whose diverse molecular, morphological and functional changes are modulated by distinct signaling mechanisms. Conditional inactivation of the signal transducer and activator of transcription 3 (*Stat3*) decreases reactive astrocyte migration after traumatic spinal cord injury [52]. Conditional inactivation of the suppressor of cytokine signaling 3 (*Socs3*) enhances reactive astrocyte migration [52]. In contrast our inducible inactivation of *p27 *resulted in Müller glial cell cycle entry and nuclear migration followed by intermediate filament upregulation (Figure 6A), but did not result in additional reactive gliosis components including hypertrophy, scar formation, edema, and loss of homeostatic and metabolic enzymes (Figure 6B). Diversity in the glial response to stress is widely documented by transcriptome-wide studies. While there is a subset of gene expression changes shared across models and CNS tissues (for example, *Gfap *and *Fos*), there are context dependent expression changes suggesting diversity and complexity in the gliotic response [14,53,54]. Gene expression data also suggest that the process of reactivity is dynamic with temporal changes clustering into at least three categories: immediate, delayed and chronic [14,53]. Thus, gene targeting and expression studies argue that reactive gliosis is context-dependent, graded and dynamic. As genetic targeting studies continue to dissect individual components of reactive gliosis, a better understanding of the functional significance of each component and its role in specific conditions will emerge.

### Transient GFAP expression in Müller glia is compatible with neuronal metabolism and function

Upregulation of the intermediate filament GFAP is arguably the most extensively described hallmark of reactive glia, yet it remains unclear whether this property is beneficial or detrimental to neuronal function and survival. The negative view regarding GFAP elevation stems from the correlation between decreased metabolic support and neuronal dysfunction with increased GFAP expression in the retina and brain [11,13,19,20,41]. For example, retinal detachment upregulates GFAP expression and concomitantly results in loss of GLUL expression and extensive derangement of glial and neuronal metabolite profiles [42]. Genetic targeting of intermediate filament expression levels (inactivation or overexpression) [16,50] and our inducible model of reactive gliosis reveal a complex role for intermediate filaments on the physiology and pathology of glial cells. While mice lacking intermediate filaments show no developmental or motor functional deficits, they display compromised blood-brain barrier, enhanced hippocampal long-term potentiation, decreased cerebellar long-term depression, white matter pathologies and demyelination [8,9,55,56]. In disease models, the absence of intermediate filaments exacerbates traumatic and toxic injury, autoimmune response, stroke, and scrapie prion infection by reducing glial hypertrophy and scarring, and compromising the ability of glial cells to osmoregulate, transport glutamate, and repair the blood-brain barrier [7-10,56]. Positive outcomes of reducing glial hypertrophy and scarring in mice lacking intermediate filaments include enhanced adult neurogenesis, axon regeneration, and neural graft survival and integration [17,57,17,25]. However, the extent to which increased regeneration potential improves functional recovery in intermediate filament deficient mice is controversial [9,10,17]. These findings have raised the question that intermediate filament upregulation plays a beneficial role in the acute stage, but prolonged upregulation interferes with neuronal survival and regeneration [56]. Supporting this view is the finding that mice transgenic for human GFAP accumulate high levels of GFAP leading to glial hypertrophy, intracytoplasmic aggregates, stress response, oxidative stress, microglial activation and neuronal dysfunction [58,59]. In contrast, transient GFAP upregulation in *p27^L-/L- ^*Müller glia was compatible with glial function (osmoregulation, transmitter recyling, radical and retinoid metabolism), neuronal transmission, and visual function. Taken together, data from mice with null, transgenic, and transient GFAP expression suggest that intermediate filament upregulation facilates the cytoarchitectural remodeling necessary for glial cells to protect the integrity of the tissue, limit the lesion site and modulate basic neuroprotective function of glial cells; but prolonged and robust intermediate filament expression can lead to glial dysfunction.

### Proliferative Müller reactive gliosis is insufficient to induce glial dysfunction, scar formation or neurogenesis

In mammals, glial cell proliferation localizes to areas of severe tissue damage after trauma, ischemia, infection, autoimmune response, or fast degenerative disease [5,6,60]. In these cases, proliferative gliosis can contribute to the formation of scars, which are hypothesized to impair neuronal function, block axonal regeneration, and interfere with tissue grafts and integration of transplanted cells [17,18]. The contribution of glial proliferation to scar formation was confirmed when proliferating astrocytes were genetically ablated after traumatic brain and spinal cord injury resulting in decreased scar formation. However, there was also impaired functional recovery that correlated with enhanced spread and persistence of inflammatory cells, failure to repair the blood-brain barrier, enhanced tissue damage, neuronal loss, and demyelination [5,6,60]. The negative outcomes from ablating proliferative astrocytes argue for the need to define the individual components of reactive gliosis that are detrimental or beneficial to neuronal function and survival. In our study, inducible Müller glial proliferation, intermediate filament upregulation and migration did not result in scar formation, glial dysfunction or neuronal deficits. The benign impact of inducible proliferative reactive gliosis in our model might be explained by the relatively low level of proliferation, but a more likely explanation is the absence of other changes associated with reactivity such as hypertrophy, decreased potassium clearance, and loss of homeostatic and metabolic enzymes [4,11,21,41]. Consequently, the extent to which neuronal cell stress, microglial reactivity, inflammatory response, blood barrier damage, or neuronal cell death contribute to the transition from supportive glia to detrimental glia needs to be determined (Figure 6B).

## Conclusions

Reactive gliosis is a ubiquitous but poorly understood hallmark of CNS and retinal pathologies. Despite the extensive characterization and manipulation of reactive gliosis, several questions remain unresolved. What regulates reactive gliosis? Is the response binary or graded? Is the response detrimental or beneficial to neuronal function and survival? Our inducible model of *p27 *inactivation exhibited three indices of glial reactivity--proliferation, intermediate filament upregulation, and migratory-like behavior--and allowed us to evaluate their intrinsic impact on retinal neuron function and survival (Figure 6A). Our metabolic, electrophysiological, and visual behavior data argue that proliferative reactive gliosis is compatible with neuronal metabolism and function, and that the final detrimental outcome of glial plasticity during degeneration or injury is determined by additional factors (Figure 6B). In combination with genetic, pharmacological or disease model approaches, the inducible model of proliferative reactive gliosis based on conditional *p27 *inactivation will be a powerful tool to dissect the factors that induce glial dysfunction or neurogenesis.

## Methods

### Animals

*p27LoxP *mice (coisogenic to 129S4) were bred to *CAGG::*CreER™ mice (Jax Stock #4453) and backcrossed 10 generations to 129S4 to generate a tamoxifen inducible p27 knockout mutation (*p27^L/L^*;CreER™) [35,36]. The promoter driving Cre-ER™ expression is a chimeric promoter of the cytomegalovirus immediate-early enhancer and chicken beta-actin promoter/enhancer (*CAGG*) known to drive a widespread expression [36]. Genotyping was performed by PCR as previously described [35,36]. Tamoxifen-treated controls are listed in Figure 1C. This research protocol was approved by the Institutional Animal Care and Use Committee of the University of Utah, and the Fred Hutchinson Cancer Research Center, and conforms to the standards in The Association for Research in Vision and Ophthalmology (ARVO) statement for the Use of Animals in Ophthalmic and Vision Research.

### Tamoxifen treatment

Tamoxifen (Sigma) was dissolved in peanut oil (Sigma) to a concentration of 5 mg·ml^-1^. 50 μg of Tamoxifen per gram of body weight (gbw) was administered to 2-month-old mice by single daily intraperitoneal injections for 5 consecutive days. All experimental and control (referred to as tamoxifen control) mice were exposed to the identical tamoxifen treatment regimen.

### BrdU administration

BrdU (Sigma) was dissolved in 0.1 M phosphate buffered saline, pH 7.4., to a concentration of 10 mg·ml^-1^. 100 μg BrdU per gbw was administered by single daily intraperitoneal injections for 3 consecutive days.

### Immunohistology

Tissue samples were prepared, processed and analyzed as described previously [61]. Table in Additional file 5 lists the antibodies used for this study. Sections were collected at the level of the optic nerve to make more direct comparisons between experimental and tamoxifen-control retinas (Additional file 1). Averages from biological replicates were pooled together to determine the total average and standard deviation. P-values were determined with unpaired Student's t tests.

### Computational molecular phenotyping (CMP)

Tissues were fixed as previously described for electron microscopy (omitting osmium) and serially sectioned at 200 nm onto 12-well HTC Cel-Line slides (Erie Scientific) ([12,21,42,62], http://prometheus.med.utah.edu/~marclab/protocols.html). Additional file 5 lists the antibodies compatible with CMP. IgG binding was visualized with silver-intensification of 1.4 nm gold granules coupled to species-specific secondary IgGs (Nanoprobes, Yaphank, NY), captured as 8-bit high-resolution (221 nm/pixel) images, mosaicked into large arrays and registered using IR-tweak software http://www.sci.utah.edu/~koshevoy/research/. CMP classification (K-means clustering and histogram analysis) was performed as previously described [42,62]. Monochrome images are density mapped and rgb images are intensity mapped (see [42]). All images were prepared in Adobe Photoshop^® ^CS3 (Adobe Systems Inc., San Jose CA).

### Mouse electroretinogram (ERG)

Full-field corneal ERGs were recorded with the universal testing and electrophysiological system UTAS E-3000 (LKC Technologies, Inc., Gaithersburg, MD). Dark adaptated mice were anesthetized by intraperitoneal injection with ketamine (100 mg/kg) and xylazine (10 mg/kg). Before recording, pupils were dilated with 2.5% phenylephrine (Akorn, Inc., Decatur, IL). Light flash intensities for scotopic ERGs ranged from -3.7 to 2.8 log cd·m^-2^. Light flash intensities for photopic ERGs ranged from -0.8 to 2.9 log cd·m^-2 ^under rod saturating background light of 1.48 log cd·m^-2^. Six or fewer flashes were averaged for each intensity level with increasing flash intervals as increasing intensity. Scotopic and photopic ERG responses of *p27^L-/L- ^*mice and age-matched tamoxifen control mice were analyzed with two-way ANOVA.

### Visual acuity test

Optomotor tracking response to moving gratings was measured using a virtual optomotor system (Opto-Motry; CerebralMechanics, Lethbridge, Alberta, Canada) as previously described [47]. The virtual cylinder was rotated at a constant speed (12°/s). On each trial an experimenter judged whether the mouse made tracking movements with its head and neck to follow the drifting grating. The spatial frequency threshold, the point at which animals no longer tracked, was obtained by incrementally increasing the spatial frequency of the grating at 100% contrast. Thresholds through each eye were measured separately by reversing the rotation of the cylinder [47].

## Authors' contributions

FVC designed research, performed research, analyzed data, and wrote manuscript. AS performed research. WDF performed research and analyzed data. LJ performed research and analyzed data. WB analyzed data. WMC performed research. MLF contributed new reagents or analytic tools. REM contributed new reagents and analytic tools, analyzed data, and edited manuscript. EML designed research, analyzed data, and edited manuscript. All authors read and approved the final manuscript.

## Supplementary Material

Additional file 1 **p27 inactivation at the periphery and intermediate filament expression in *p27^L-/L- ^*retina**. (*A*-*H*) Fluoresence microscopy of retinas from tamoxifen control and *p27^L-/L- ^*mice. Time after inactivation is specified for each panel. (*C*) Inactivation efficiency was measured by comparing the number of p27^+ ^cells colocalizing with the glial nuclear marker SOX9. Data are expressed as the mean ± SD. (*D*) Mosaic of 20× magnification tiles illustrates the location of the central and peripheral retinal portions, and the optic nerve (ON). Abbreviations: p27, cyclin-dependent kinase inhibitor CDKN1B; GFAP, glial fibrillary acidic protein; and SLC1A3, solute carrier family 1, member 3 (also known as GLAST). Click here for file

Additional file 2 **Upregulation of proliferative markers in Müller glia exclusive to *p27^L-/L- ^*retina**. (*A*-*G*) Fluoresence microscopy of retinas from tamoxifen control and *p27^L-/L- ^*mice. Time after inactivation is specified for each panel. Abbreviations: ONL, outer nuclear layer; INL, inner nuclear layer; RGC, retinal ganglion cell layer; GLUL, glutamine synthetase; pHH3, phospho-histone H3; PCNA, proliferating cell nuclear antigen. SOX9, SRY-box containing gene 9; MCM6, minichromosome maintenance complex component 6; and DAPI, 4', 6-diamidino-2-phenylindole. Click here for file

Additional file 3 **Ultrathin immunohistology of *p27^L-/L- ^*retina**. (*A*-*D*) Immunohistology and analyses based off epon-embedded, ultrathin sections (200 nm) and silver intensification. (*A *and *B*) Radial sections probed against GLUL. Somas and stalks were numbered in green and red, respectively. (*C*) Quantitation of GLUL^+ ^somas and stalks in oblique sections. (*D*) Potential model of morphological changes in dividing Müller glia and daughter cell. (*E*) Radial sections probed against GABA. Abbreviations: ONL, outer nuclear layer; INL, inner nuclear layer; RGC, retinal ganglion cell layer; GLUL, glutamine synthetase; OPL, outer plexus layer; BCL, Bipolar cell layer; MCL, Müller cell layer; ACL, amacrine cell layer; IPL, inner plexus layer; and GABA, γ-Aminobutyric acid. Click here for file

Additional file 4 **Immunohistology in *p27^L-/L- ^*retina and distal optic nerve**. (*A*-*F*) Fluoresence microscopy of retinas (*A-D*) or distal optic nerve (*E, F*) from tamoxifen control and *p27^L-/L- ^*mice. Time after inactivation is specified for each panel. CD44 labels the Müller glia microvilli at the outer limiting membrane. Abbreviations: KIR4.1, potassium channel, inward rectifier 4.1. Click here for file

Additional file 5 **Immunoreagents and their compatibility with computational metabolic profiling (CMP) or immunocytochemistry (ICC)**. Table listing immunoreagents. Click here for file

## References

[B1] Eddleston M Mucke L Molecular profile of reactive astrocytes--implications for their role in neurologic disease Neuroscience 1993 54 1 15 36 10.1016/0306-4522(93)90380-X 8515840 PMC7130906

[B2] Reier PJ Gliosis following CNS injury: The anatomy of astrocyte scars and their influences on axonal elongation 1986 Orlando: Academic Press

[B3] Ridet JL Malhotra SK Privat A Gage FH Reactive astrocytes: cellular and molecular cues to biological function Trends Neurosci 1997 20 12 570 577 10.1016/S0166-2236(97)01139-9 9416670

[B4] Sarthy V Ripps H The retinal Müller cell: structure and function 2001 New York: Kluwer Academic/Plenum Publishers

[B5] Bush TG Puvanachandra N Horner CH Polito A Ostenfeld T Svendsen CN Mucke L Johnson MH Sofroniew MV Leukocyte infiltration, neuronal degeneration, and neurite outgrowth after ablation of scar-forming, reactive astrocytes in adult transgenic mice Neuron 1999 23 2 297 308 10.1016/S0896-6273(00)80781-3 10399936

[B6] Faulkner JR Herrmann JE Woo MJ Tansey KE Doan NB Sofroniew MV Reactive astrocytes protect tissue and preserve function after spinal cord injury J Neurosci 2004 24 9 2143 2155 10.1523/JNEUROSCI.3547-03.2004 14999065 PMC6730429

[B7] Gomi H Yokoyama T Fujimoto K Ikeda T Katoh A Itoh T Itohara S Mice devoid of the glial fibrillary acidic protein develop normally and are susceptible to scrapie prions Neuron 1995 14 1 29 41 10.1016/0896-6273(95)90238-4 7826639

[B8] Li L Lundkvist A Andersson D Wilhelmsson U Nagai N Pardo AC Nodin C Stahlberg A Aprico K Larsson K Protective role of reactive astrocytes in brain ischemia J Cereb Blood Flow Metab 2008 28 3 468 481 10.1038/sj.jcbfm.9600546 17726492

[B9] Liedtke W Edelmann W Bieri PL Chiu FC Cowan NJ Kucherlapati R Raine CS GFAP is necessary for the integrity of CNS white matter architecture and long-term maintenance of myelination Neuron 1996 17 4 607 615 10.1016/S0896-6273(00)80194-4 8893019

[B10] Otani N Nawashiro H Fukui S Ooigawa H Ohsumi A Toyooka T Shima K Gomi H Brenner M Enhanced hippocampal neurodegeneration after traumatic or kainate excitotoxicity in GFAP-null mice J Clin Neurosci 2006 13 9 934 938 10.1016/j.jocn.2005.10.018 17085299

[B11] Giaume C Kirchhoff F Matute C Reichenbach A Verkhratsky A Glia: the fulcrum of brain diseases Cell Death Differ 2007 14 7 1324 1335 10.1038/sj.cdd.4402144 17431421

[B12] Marc RE Jones BW Watt CB Vazquez-Chona F Vaughan DK Organisciak DT Extreme retinal remodeling triggered by light damage: implications for age related macular degeneration Mol Vis 2008 14 782 806 18483561 PMC2375357

[B13] Croisier E Graeber MB Glial degeneration and reactive gliosis in alpha-synucleinopathies: the emerging concept of primary gliodegeneration Acta Neuropathol 2006 112 5 517 530 10.1007/s00401-006-0119-z 16896905

[B14] Di Giovanni S Movsesyan V Ahmed F Cernak I Schinelli S Stoica B Faden AI Cell cycle inhibition provides neuroprotection and reduces glial proliferation and scar formation after traumatic brain injury Proc Natl Acad Sci USA 2005 102 23 8333 8338 10.1073/pnas.0500989102 15923260 PMC1149422

[B15] Lewis GP Fisher SK Up-regulation of glial fibrillary acidic protein in response to retinal injury: its potential role in glial remodeling and a comparison to vimentin expression Int Rev Cytol 2003 230 263 290 14692684 10.1016/s0074-7696(03)30005-1

[B16] Pekny M Nilsson M Astrocyte activation and reactive gliosis Glia 2005 50 4 427 434 10.1002/glia.20207 15846805

[B17] Menet V Gimenez y Ribotta M Chauvet N Drian MJ Lannoy J Colucci-Guyon E Privat A Inactivation of the glial fibrillary acidic protein gene, but not that of vimentin, improves neuronal survival and neurite growth by modifying adhesion molecule expression J Neurosci 2001 21 16 6147 6158 11487638 10.1523/JNEUROSCI.21-16-06147.2001 PMC6763158

[B18] Rudge JS Silver J Inhibition of neurite outgrowth on astroglial scars in vitro J Neurosci 1990 10 11 3594 3603 2230948 10.1523/JNEUROSCI.10-11-03594.1990 PMC6570102

[B19] DiLoreto D Ison JR Bowen GP Cox C del Cerro M A functional analysis of the age-related degeneration in the Fischer 344 rat Curr Eye Res 1995 14 4 303 310 10.3109/02713689509033530 7606916

[B20] DiLoreto DA Martzen MR del Cerro C Coleman PD del Cerro M Muller cell changes precede photoreceptor cell degeneration in the age-related retinal degeneration of the Fischer 344 rat Brain Res 1995 698 1-2 1 14 10.1016/0006-8993(95)00647-9 8581466

[B21] Marc RE Murry RF Fisher SK Linberg KA Lewis GP Amino acid signatures in the detached cat retina Invest Ophthalmol Vis Sci 1998 39 9 1694 1702 9699559

[B22] Byrnes KR Faden AI Role of cell cycle proteins in CNS injury Neurochem Res 2007 32 10 1799 1807 10.1007/s11064-007-9312-2 17404835

[B23] Crockett DP Burshteyn M Garcia C Muggironi M Casaccia-Bonnefil P Number of oligodendrocyte progenitors recruited to the lesioned spinal cord is modulated by the levels of the cell cycle regulatory protein p27Kip-1 Glia 2005 49 2 301 308 10.1002/glia.20111 15472992

[B24] Dyer MA Cepko CL Control of Muller glial cell proliferation and activation following retinal injury Nat Neurosci 2000 3 9 873 880 10.1038/78774 10966617

[B25] Koguchi K Nakatsuji Y Nakayama K Sakoda S Modulation of astrocyte proliferation by cyclin-dependent kinase inhibitor p27(Kip1) Glia 2002 37 2 93 104 10.1002/glia.10017 11754208

[B26] Levine EM Close J Fero M Ostrovsky A Reh TA p27(Kip1) regulates cell cycle withdrawal of late multipotent progenitor cells in the mammalian retina Dev Biol 2000 219 2 299 314 10.1006/dbio.2000.9622 10694424

[B27] Shen A Liu Y Zhao J Qin J Shi S Chen M Gao S Xiao F Lu Q Cheng C Temporal-spatial expressions of p27kip1 and its phosphorylation on Serine-10 after acute spinal cord injury in adult rat: Implications for post-traumatic glial proliferation Neurochem Int 2008 52 6 1266 1275 10.1016/j.neuint.2008.01.011 18319192

[B28] Lopez-Sanchez E Frances-Munoz E Chaques V Sanchez-Benavent ML Menezo JL Optic nerve alterations in P27(Kip1) knockout mice Eur J Ophthalmol 2007 17 3 377 382 17534820 10.1177/112067210701700317

[B29] Cunningham JJ Levine EM Zindy F Goloubeva O Roussel MF Smeyne RJ The cyclin-dependent kinase inhibitors p19(Ink4d) and p27(Kip1) are coexpressed in select retinal cells and act cooperatively to control cell cycle exit Mol Cell Neurosci 2002 19 3 359 374 10.1006/mcne.2001.1090 11906209

[B30] Besson A Dowdy SF Roberts JM CDK inhibitors: cell cycle regulators and beyond Dev Cell 2008 14 2 159 169 10.1016/j.devcel.2008.01.013 18267085

[B31] Frank CL Tsai LH Alternative functions of core cell cycle regulators in neuronal migration, neuronal maturation, and synaptic plasticity Neuron 2009 62 3 312 326 10.1016/j.neuron.2009.03.029 19447088 PMC2757047

[B32] Nakayama K Ishida N Shirane M Inomata A Inoue T Shishido N Horii I Loh DY Nakayama K Mice lacking p27(Kip1) display increased body size, multiple organ hyperplasia, retinal dysplasia, and pituitary tumors Cell 1996 85 5 707 720 10.1016/S0092-8674(00)81237-4 8646779

[B33] Tokita-Ishikawa Y Wakusawa R Abe T Evaluation of Retinal Degeneration in P27KIP1 Null Mouse Adv Exp Med Biol 2010 664 467 471 10.1007/978-1-4419-1399-9_53 20238048

[B34] Yoshida K Kase S Nakayama K Nagahama H Harada T Ikeda H Harada C Imaki J Ohgami K Shiratori K Distribution of p27(KIP1), cyclin D1, and proliferating cell nuclear antigen after retinal detachment Graefes Arch Clin Exp Ophthalmol 2004 242 5 437 441 10.1007/s00417-004-0861-7 15029501

[B35] Chien WM Rabin S Macias E Miliani de Marval PL Garrison K Orthel J Rodriguez-Puebla M Fero ML Genetic mosaics reveal both cell-autonomous and cell-nonautonomous function of murine p27Kip1 Proc Natl Acad Sci USA 2006 103 11 4122 4127 10.1073/pnas.0509514103 16537495 PMC1449657

[B36] Hayashi S McMahon AP Efficient recombination in diverse tissues by a tamoxifen-inducible form of Cre: a tool for temporally regulated gene activation/inactivation in the mouse Dev Biol 2002 244 2 305 318 10.1006/dbio.2002.0597 11944939

[B37] Nayfield SG Gorin MB Tamoxifen-associated eye disease. A review J Clin Oncol 1996 14 3 1018 1026 8622006 10.1200/JCO.1996.14.3.1018

[B38] Slezak M Goritz C Niemiec A Frisen J Chambon P Metzger D Pfrieger FW Transgenic mice for conditional gene manipulation in astroglial cells Glia 2007 55 15 1565 1576 10.1002/glia.20570 17823970

[B39] Ekstrom P Sanyal S Narfstrom K Chader GJ van Veen T Accumulation of glial fibrillary acidic protein in Muller radial glia during retinal degeneration Invest Ophthalmol Vis Sci 1988 29 9 1363 1371 3417421

[B40] Shaw G Weber K The structure and development of the rat retina: an immunofluorescence microscopical study using antibodies specific for intermediate filament proteins Eur J Cell Biol 1983 30 2 219 232 11596496

[B41] D'Ambrosio R The role of glial membrane ion channels in seizures and epileptogenesis Pharmacol Ther 2004 103 2 95 108 10.1016/j.pharmthera.2004.05.004 15369678

[B42] Marc RE Murry RF Basinger SF Pattern recognition of amino acid signatures in retinal neurons J Neurosci 1995 15 7 Pt 2 5106 5129 7623139 10.1523/JNEUROSCI.15-07-05106.1995 PMC6577898

[B43] Winkler BS Kapousta-Bruneau N Arnold MJ Green DG Effects of inhibiting glutamine synthetase and blocking glutamate uptake on b-wave generation in the isolated rat retina Vis Neurosci 1999 16 2 345 353 10.1017/S095252389916214X 10367968 PMC1885536

[B44] Kofuji P Ceelen P Zahs KR Surbeck LW Lester HA Newman EA Genetic inactivation of an inwardly rectifying potassium channel (Kir4.1 subunit) in mice: phenotypic impact in retina J Neurosci 2000 20 15 5733 5740 10908613 10.1523/JNEUROSCI.20-15-05733.2000 PMC2410027

[B45] Witkovsky P Dudek FE Ripps H Slow PIII component of the carp electroretinogram J Gen Physiol 1975 65 2 119 134 10.1085/jgp.65.2.119 1117278 PMC2214870

[B46] Pinto LH Invergo B Shimomura K Takahashi JS Troy JB Interpretation of the mouse electroretinogram Doc Ophthalmol 2007 115 3 127 136 10.1007/s10633-007-9064-y 17636411 PMC3786689

[B47] Prusky GT Alam NM Beekman S Douglas RM Rapid quantification of adult and developing mouse spatial vision using a virtual optomotor system Invest Ophthalmol Vis Sci 2004 45 12 4611 4616 10.1167/iovs.04-0541 15557474

[B48] Close JL Liu J Gumuscu B Reh TA Epidermal growth factor receptor expression regulates proliferation in the postnatal rat retina Glia 2006 54 2 94 104 10.1002/glia.20361 16710850

[B49] Ooto S Akagi T Kageyama R Akita J Mandai M Honda Y Takahashi M Potential for neural regeneration after neurotoxic injury in the adult mammalian retina Proc Natl Acad Sci USA 2004 101 37 13654 13659 10.1073/pnas.0402129101 15353594 PMC518808

[B50] Messing A Brenner M GFAP: functional implications gleaned from studies of genetically engineered mice Glia 2003 43 1 87 90 10.1002/glia.10219 12761871

[B51] Sofroniew MV Reactive astrocytes in neural repair and protection Neuroscientist 2005 11 5 400 407 10.1177/1073858405278321 16151042

[B52] Okada S Nakamura M Katoh H Miyao T Shimazaki T Ishii K Yamane J Yoshimura A Iwamoto Y Toyama Y Conditional ablation of Stat3 or Socs3 discloses a dual role for reactive astrocytes after spinal cord injury Nat Med 2006 12 7 829 834 10.1038/nm1425 16783372

[B53] Vazquez-Chona F Song BK Geisert EE Jr Temporal changes in gene expression after injury in the rat retina Invest Ophthalmol Vis Sci 2004 45 8 2737 2746 10.1167/iovs.03-1047 15277499 PMC2821791

[B54] Zhang Y Barres BA Astrocyte heterogeneity: an underappreciated topic in neurobiology Curr Opin Neurobiol 2010 20 5 588 594 10.1016/j.conb.2010.06.005 20655735

[B55] McCall MA Gregg RG Behringer RR Brenner M Delaney CL Galbreath EJ Zhang CL Pearce RA Chiu SY Messing A Targeted deletion in astrocyte intermediate filament (Gfap) alters neuronal physiology Proc Natl Acad Sci USA 1996 93 13 6361 6366 10.1073/pnas.93.13.6361 8692820 PMC39027

[B56] Pekny M Wilhelmsson U Bogestal YR Pekna M The role of astrocytes and complement system in neural plasticity Int Rev Neurobiol 2007 82 95 111 17678957 10.1016/S0074-7742(07)82005-8

[B57] Kinouchi R Takeda M Yang L Wilhelmsson U Lundkvist A Pekny M Chen DF Robust neural integration from retinal transplants in mice deficient in GFAP and vimentin Nat Neurosci 2003 6 8 863 868 10.1038/nn1088 12845328

[B58] Messing A Head MW Galles K Galbreath EJ Goldman JE Brenner M Fatal encephalopathy with astrocyte inclusions in GFAP transgenic mice Am J Pathol 1998 152 2 391 398 9466565 PMC1857948

[B59] Quinlan RA Brenner M Goldman JE Messing A GFAP and its role in Alexander disease Exp Cell Res 2007 313 10 2077 2087 10.1016/j.yexcr.2007.04.004 17498694 PMC2702672

[B60] Sofroniew MV Molecular dissection of reactive astrogliosis and glial scar formation Trends Neurosci 2009 32 12 638 647 10.1016/j.tins.2009.08.002 19782411 PMC2787735

[B61] Vazquez-Chona FR Clark AM Levine EM Rlbp1 promoter drives robust Muller glial GFP expression in transgenic mice Invest Ophthalmol Vis Sci 2009 50 8 3996 4003 10.1167/iovs.08-3189 19324864

[B62] Marc RE Jones BW Molecular phenotyping of retinal ganglion cells J Neurosci 2002 22 2 413 427 11784786 10.1523/JNEUROSCI.22-02-00413.2002 PMC6758675

