# Supplementary figures and images for: Proliferative reactive gliosis is compatible with glial metabolic support and neuronal function
Source: BMC Neurosci. 2011 Oct 10;12:98. doi: 10.1186/1471-2202-12-98 (PMC3203081; doi:10.1186/1471-2202-12-98)

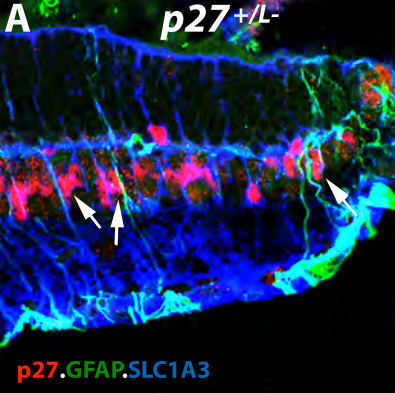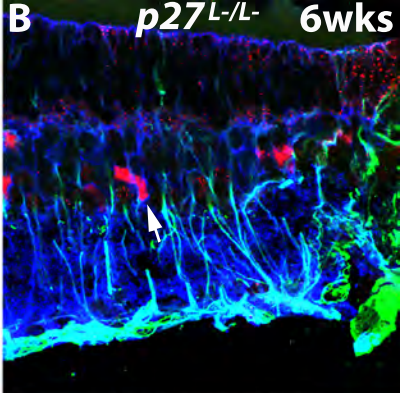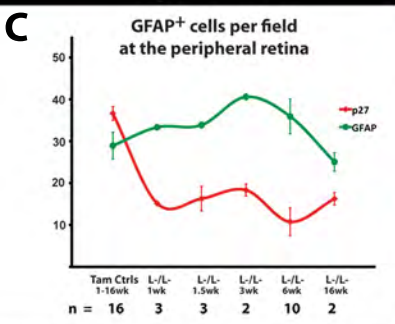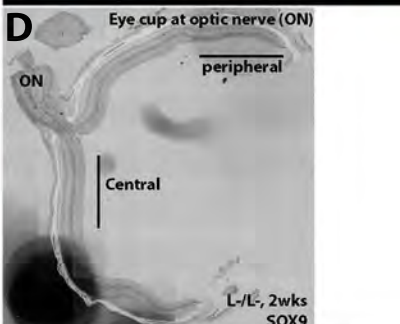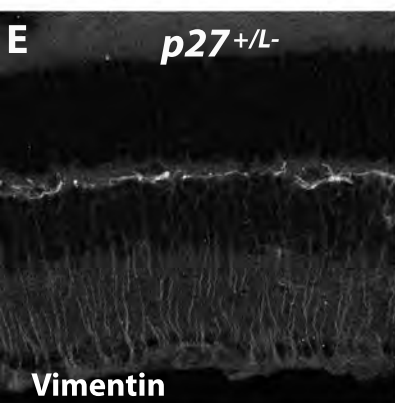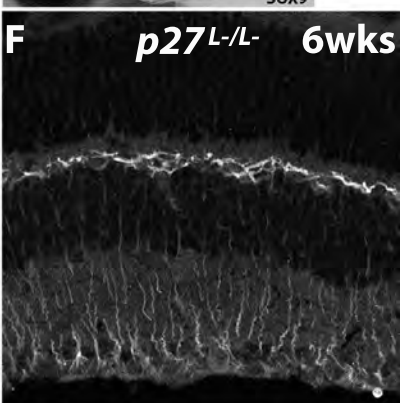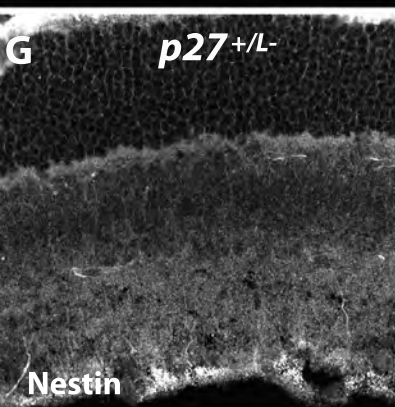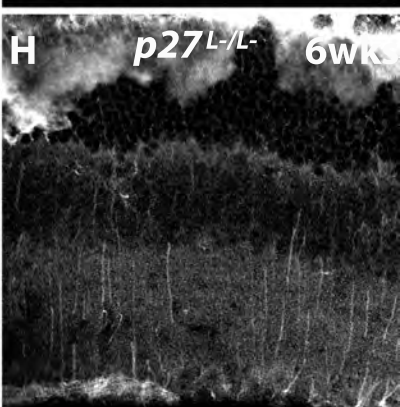

Supplement: Additional file 1 — p27 inactivation at the periphery and intermediate filament expression in p27L-/L- retina. (A-H) Fluoresence microscopy of retinas from tamoxifen control and p27L-/L- mice. Time after inactivation is specified for each panel. (C) Inactivation efficiency was measured by comparing the number of p27+ cells colocalizing with the glial nuclear marker SOX9. Data are expressed as the mean ± SD. (D) Mosaic of 20× magnification tiles illustrates the location of the central and peripheral retinal portions, and the optic nerve (ON). Abbreviations: p27, cyclin-dependent kinase inhibitor CDKN1B; GFAP, glial fibrillary acidic protein; and SLC1A3, solute carrier family 1, member 3 (also known as GLAST). [file 1471-2202-12-98-S1.PDF]

*p27<sup>L-/L-</sup>*, 1.5 wk

*p27<sup>L-/L-</sup>*, 1.5 wk

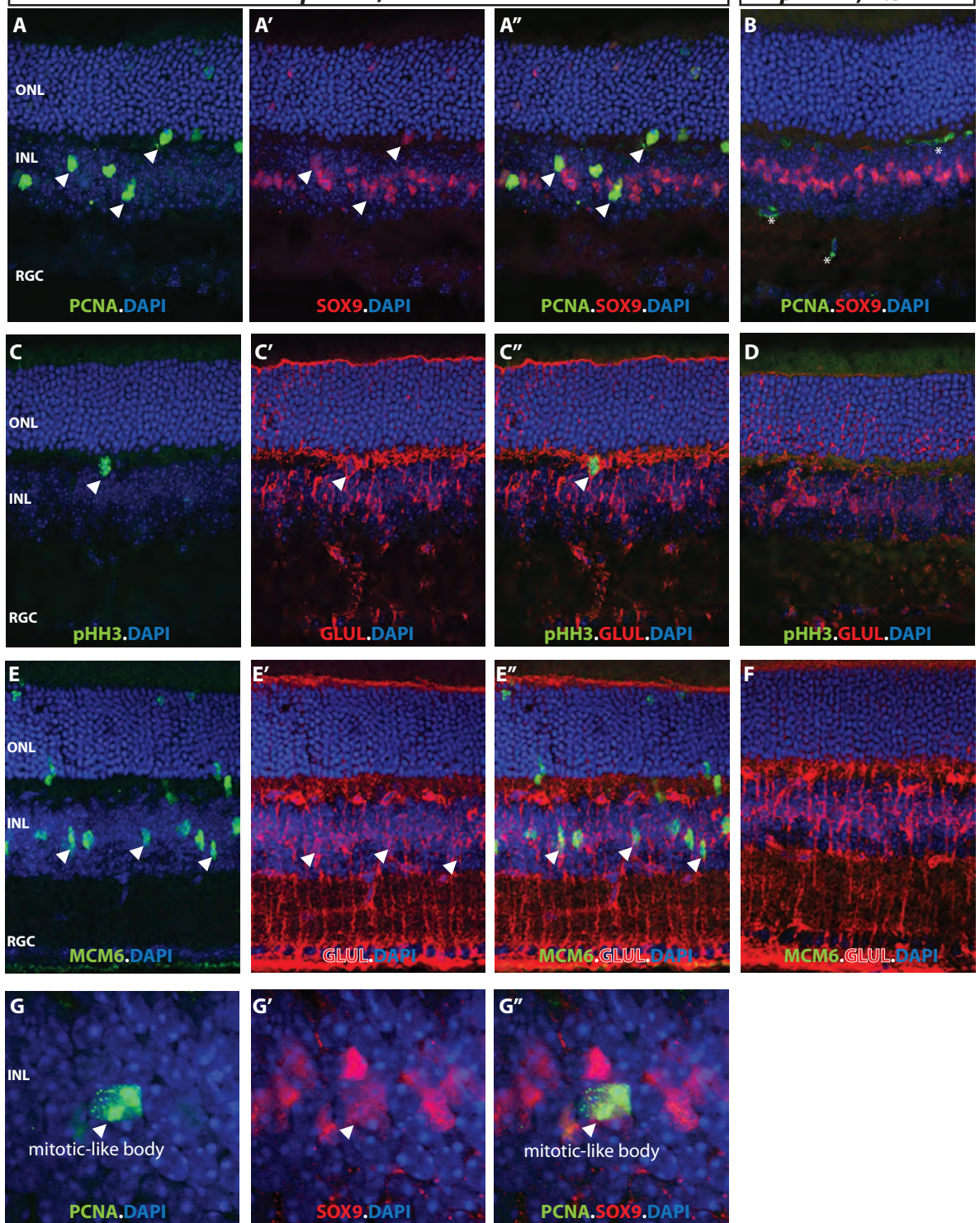

Supplement: Additional file 2 — Upregulation of proliferative markers in Müller glia exclusive to p27L-/L- retina. (A-G) Fluoresence microscopy of retinas from tamoxifen control and p27L-/L- mice. Time after inactivation is specified for each panel. Abbreviations: ONL, outer nuclear layer; INL, inner nuclear layer; RGC, retinal ganglion cell layer; GLUL, glutamine synthetase; pHH3, phospho-histone H3; PCNA, proliferating cell nuclear antigen. SOX9, SRY-box containing gene 9; MCM6, minichromosome maintenance complex component 6; and DAPI, 4', 6-diamidino-2-phenylindole. [file 1471-2202-12-98-S2.PDF]

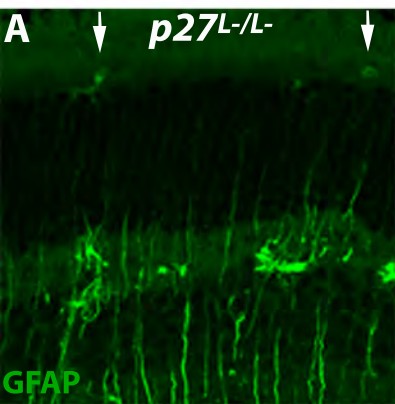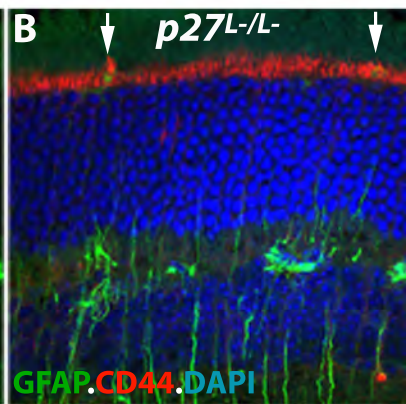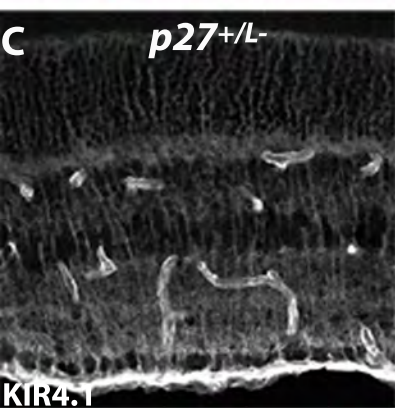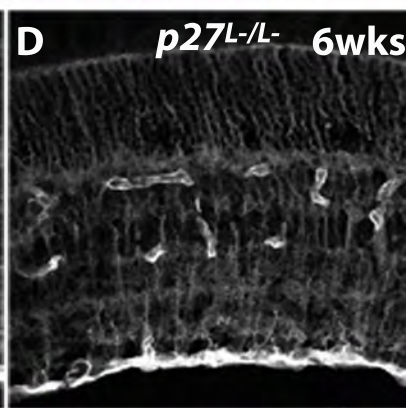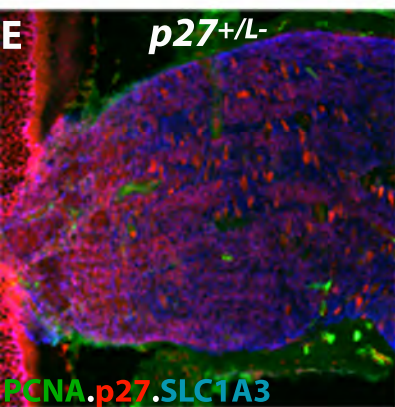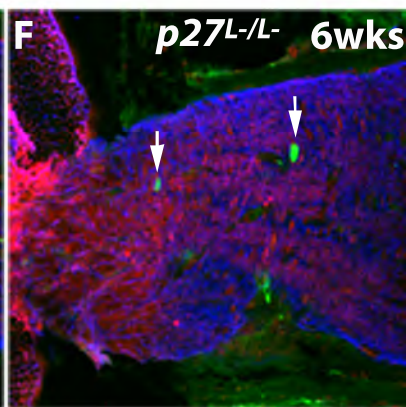

Supplement: Additional file 4 — Immunohistology in p27L-/L- retina and distal optic nerve. (A-F) Fluoresence microscopy of retinas (A-D) or distal optic nerve (E, F) from tamoxifen control and p27L-/L- mice. Time after inactivation is specified for each panel. CD44 labels the Müller glia microvilli at the outer limiting membrane. Abbreviations: KIR4.1, potassium channel, inward rectifier 4.1. [file 1471-2202-12-98-S4.PDF]
