# Supplementary material for: Proliferative reactive gliosis is compatible with glial metabolic support and neuronal function
Source: BMC Neurosci. 2011 Oct 10;12:98. doi: 10.1186/1471-2202-12-98 (PMC3203081; doi:10.1186/1471-2202-12-98)

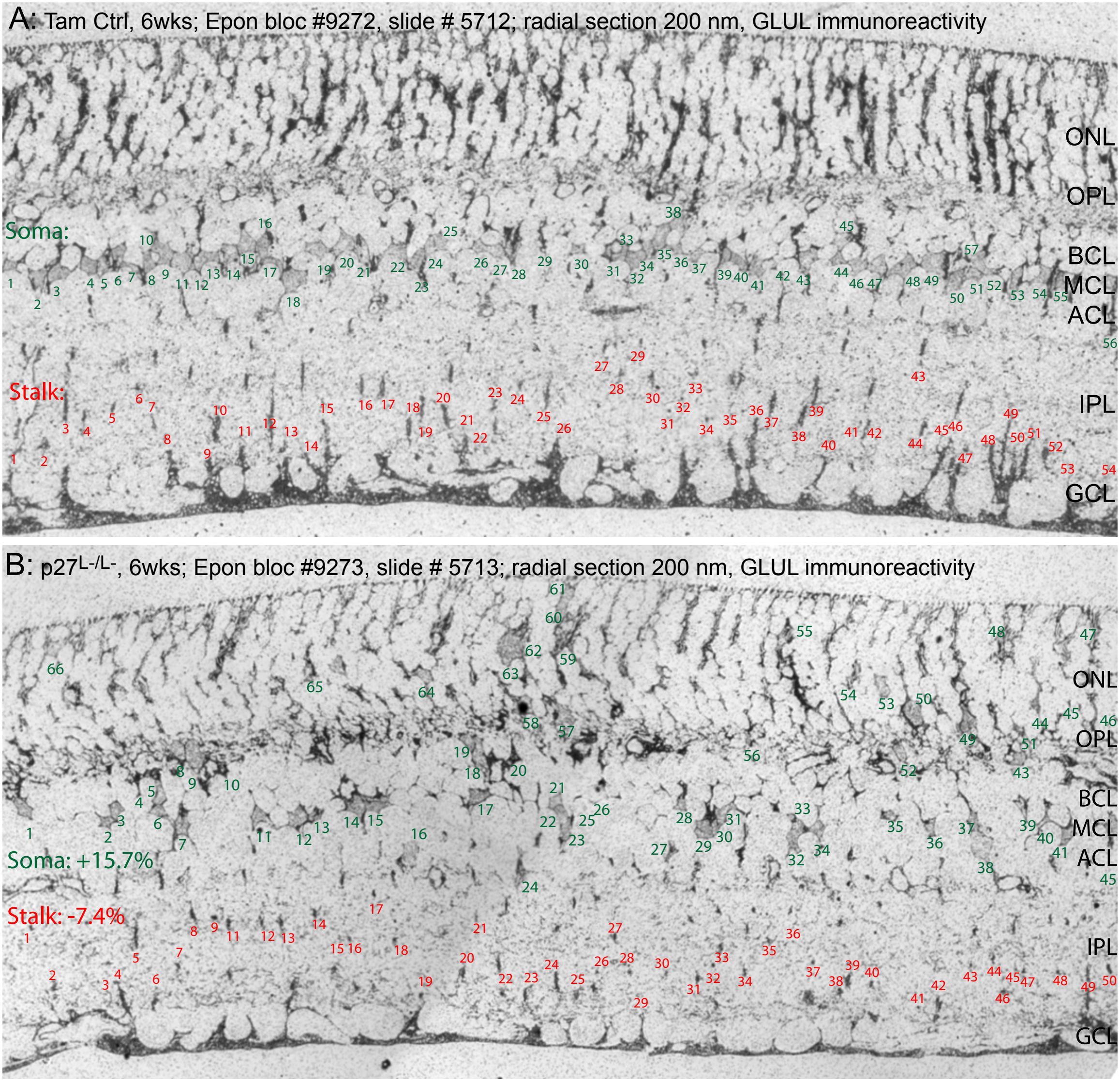

C

GLUL+ somas and stalks in oblique sections

Retinal layer	TamCtrl	p27L-/L-
Outer nuclear (ONL)	0	185
Outer plexus (OPL)	0	89
Bipolar cell (BCL)	23	132
Müller cell (MCL)	568	257
Amacrine cell (ACL)	23	16
Inner plexus (IPL)	0	0
Total somas	614	679
Soma % change		11%
Stalk density per 50µx50µ	54±3	41±5
Stalk % change		-23%
Bloc ID	9272	9273
Slide ID	6107	6108

D: Potential model of morphological changes in dividing Müller glia and daughter cell

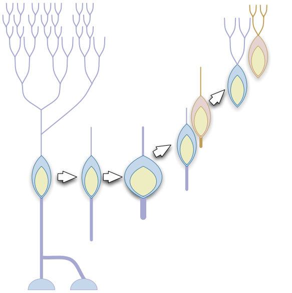

E: GABA distribution

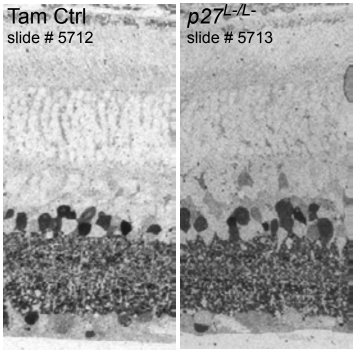

Supplement: Additional file 3 — Ultrathin immunohistology of p27L-/L- retina. (A-D) Immunohistology and analyses based off epon-embedded, ultrathin sections (200 nm) and silver intensification. (A and B) Radial sections probed against GLUL. Somas and stalks were numbered in green and red, respectively. (C) Quantitation of GLUL+ somas and stalks in oblique sections. (D) Potential model of morphological changes in dividing Müller glia and daughter cell. (E) Radial sections probed against GABA. Abbreviations: ONL, outer nuclear layer; INL, inner nuclear layer; RGC, retinal ganglion cell layer; GLUL, glutamine synthetase; OPL, outer plexus layer; BCL, Bipolar cell layer; MCL, Müller cell layer; ACL, amacrine cell layer; IPL, inner plexus layer; and GABA, γ-Aminobutyric acid. [file 1471-2202-12-98-S3.PDF]
