# Supplementary material for: Proliferative reactive gliosis is compatible with glial metabolic support and neuronal function
Source: BMC Neurosci. 2011 Oct 10;12:98. doi: 10.1186/1471-2202-12-98 (PMC3203081; doi:10.1186/1471-2202-12-98)
Supplement: Additional file 5 — Immunoreagents and their compatibility with computational metabolic profiling (CMP) or immunocytochemistry (ICC). Table listing immunoreagents. [file 1471-2202-12-98-S5.DOCX]

**Table**. Immunoreagents and their compatibility with computational metabolic profiling (CMP) or immunocytochemistry (ICC)

| ***Antibody target or dye*** | ***CMP*** | ***ICC*** | ***Host*** | ***Source*** |
| --- | --- | --- | --- | --- |
| 4',6-diamidino-2-phenylindole (DAPI) | ✓ | ✓ | - | Sigma |
| L-arginine | ✓ |  | Rabbit | Robert Marc (University of Utah) |
| L-aspartate | ✓ |  | Rabbit | Signature Immunologics |
| Bromodeoxyuridine (BrdU) |  | ✓ | Rat | AbD Serotec |
| CD44 | ✓ |  | Rat | DHSB |
| p27KIP1 (CDKN1B) |  | ✓ | Rabbit | Epitomics |
| p27KIP1 (CDKN1B) |  | ✓ | Mouse | BD transduction |
| Glial fibrillary acidic protein (GFAP) | ✓ | ✓ | Mouse | Sigma |
| Glial fibrillary acidic protein (GFAP) | ✓ | ✓ | Rabbit | Dako |
| Glial fibrillary acidic protein (GFAP) |  | ✓ | Chicken | Abcam |
| Gamma-aminobutyric acid (GABA) | ✓ |  | Rabbit | Signature Immunologics |
| L-glutamate | ✓ |  | Rabbit | Signature Immunologics |
| Glutamine | ✓ |  | Rabbit | Signature Immunologics |
| Glutamine synthetase (GLUL) | ✓ | ✓ | Mouse | BD transduction |
| Glutathione (GSH) | ✓ |  | Rabbit | Signature Immunologics |
| Minichromosome maintenance complex component 6 (MCM6) |  | ✓ | Goat | Santa Cruz |
| Nestin (NES) |  | ✓ | Mouse | DHSB |
| Orthodenticle homeobox 2 (OTX2) |  | ✓ | Goat | Santa Cruz |
| Proliferating cell nuclear antigen (PCNA) |  | ✓ | Mouse | DAKO |
| Phospho-histone H3 (pHH3) |  | ✓ | Rabbit | Upstate |
| Potassium Channel, Inward Rectifier Kir4.1 |  | ✓ | Rabbit | Chemicon |
| PKC-alpha |  | ✓ | Rabbit | Sigma |
| Recoverin |  | ✓ | Rabbit | Chemicon |
| Retinaldehyde binding protein 1 (RLBP1/CRALBP) | ✓ | ✓ | Rabbit | John Saari (University of Washington) |
| Solute carrier family 1, member 3 (SLC1A3/GLAST) |  | ✓ | Guinea pig | Chemicon |
| SRY-box containing gene 9 (SOX9) |  | ✓ | Rabbit | Chemicon |
| Visual system homeobox 2 (VSX2/CHX10) |  | ✓ | Sheep | Exalpha Biologicals |
| Vimentin |  | ✓ | Goat | Chemicon |
